# Impact of the TiO_2_ Nanosolution Concentration on Heat Transfer Enhancement of the Twin Impingement Jet of a Heated Aluminum Plate

**DOI:** 10.3390/mi10030176

**Published:** 2019-03-07

**Authors:** Mahir Faris Abdullah, Rozli Zulkifli, Zambri Harun, Shahrir Abdullah, Wan Aizon Wan Ghopa, Asmaa Soheil Najm, Noor Humam Sulaiman

**Affiliations:** 1 Department of Mechanical and Materials Engineering, Universiti Kebangsaan Malaysia, Bangi 43600, Selangor, Malaysia; zambri@ukm.edu.my (Z.H.); shahrir@ukm.edu.my (S.A.); waizon@ukm.edu.my (W.A.W.G.); nora.humam@gmail.com (N.H.S.); 2 Department of Electrical and Electronic Engineering, Universiti Kebangsaan Malaysia, Bangi 43600, Selangor, Malaysia; asmaa.soheil@yahoo.com

**Keywords:** TiO_2_ nanoparticles, nano coating, impingement jet, heat transfer, Nusselt number

## Abstract

Here, the researchers carried out an experimental analysis of the effect of the TiO_2_ nanosolution concentration on the heat transfer of the twin jet impingement on an aluminum plate surface. We used three different heat transfer enhancement processes. We considered the TiO_2_ nanosolution coat, aluminum plate heat sink, and a twin jet impingement system. We also analyzed several other parameters like the nozzle spacing, nanosolution concentration, and the nozzle-to-plate distance and noted if these parameters could increase the heat transfer rate of the twin jet impingement system on a hot aluminum surface. The researchers prepared different nanosolutions, which consisted of varying concentrations, and coated them on the metal surface. Thereafter, we carried out an X-ray diffraction (XRD) and a Field Emission Scanning Electron Microscopy (FESEM) analysis for determining the structure and the homogeneous surface coating of the nanosolutions. This article also studied the different positions of the twin jets for determining the maximal Nusselt number (Nu). The researchers analyzed all the results and noted that the flow structure of the twin impingement jets at the interference zone was the major issue affecting the increase in the heat transfer rate. The combined influence of the spacing and nanoparticle concentration affected the flow structure, and therefore the heat transfer properties, wherein the Reynolds number (1% by volume concentration) maximally affected the Nusselt number. This improved the performance of various industrial and engineering applications. Hypothesis: Nusselt number was affected by the ratio of the nanoparticle size to the surface roughness. Heat transfer characteristics could be improved if the researchers selected an appropriate impingement system and selected the optimal levels of other factors. The surface coating with the TiO_2_ nanosolution also positively affected the heat transfer rate.

## 1. Introduction

In the past few years, various researchers have found it challenging to solve the heat transfer-related problems in the engineering systems. Very few approaches have been used for solving these challenges. In one such approach, the heat transfer could be increased by improving the performance of the impingement jet system [1,2,3,4]. Studies also noted that the nanofluids were a feasible coolant that improved the performance efficiency of several engineering devices [5]. The electronic devices dissipate a lot of heat during their operation, which can directly affect their performance efficiency.

In the 1970s and 80s, ultra-fine particles with a size <100 nm, were known as nanoparticles. The researchers mixed the nanoparticles with base fluids like water, oil, or ethylene glycol for improving their properties [6]. Nanofluid refers to the mixture of the nanoparticles and the base fluid, and it is used in various heat transfer applications. Some studies used titanium oxide nanofluids in various heat transfer applications. TiO_2_ nanofluids displayed better thermal properties in comparison to simple liquids, and hence, were used as an alternative for simple liquids in different heat transfer systems like heat sinks, heat exchangers, and car radiators [7]. The studies were categorized into three classes: experimental, analytical, and numerical [8,9,10]. The nanoparticle technique helped in improving the heat transfer and the energy efficiency of various thermal systems. Thereafter, the nanofluids were used for developing cooling fluids that could be used in many applications such as refrigeration, microchip cooling, engine cooling, electronic circuit cooling, nuclear cooling systems, surface coating, thermal storage, improvement of heat transfer exchange, biomedical applications, environmental remediation, petroleum industry, transportation, defense and space applications, inkjet printing, fuel additives, and lubricants [11]. Researchers investigated the effect of different TiO_2_ nanoparticle concentrations for improving the heat transfer rate with the help of the twin impingement jet mechanism. Thereafter, the researchers studied the effect of the nanoparticle-coated aluminum surface on the Nusselt number. We, the authors, also observed the effect of the coated surface solution on the twin impingement jet process since this technique influenced the heat transfer rate of the various engineering devices. To the best of our knowledge, there is no information regarding such systems in the literature; the impact of using nanocoating in the impingement technique has not been investigated before.

The use of titanium dioxide (TiO_2_) is common in various applications such as heat transfer due to its excellent chemical and physical stability. Moreover, the TiO_2_ particles are cheap and commercially available. Another purpose of using TiO_2_ nanoparticles was their being suspended in conventional fluids, which are extensively used in different forms of heat exchangers, including circular tubes [12,13], double tubes [14,15,16], and shell and tubes [17].

### 1.1. Convection Heat Transfer of the TiO_2_ Nanoparticle Application

An increased heat transfer rate was considered to be the most vital necessity of all applications. Nanosolutions can improve the heat transfer rate and can significantly improve the thermal fluid-based applications. A higher convective heat transfer coefficient (HTC) compared to the thermal conductivity was a direct indicator of the heat exchange devices. Some changes that occurred in the physical properties of these devices, like in the viscosity, density, and the specific heat, led to a significant increase in the HTC compared to the increase in the thermal conductivity rate in several experiments. The results indicated that a majority of the studies were experimental and showed an increase in the HTC [18]. Furthermore, the results also showed that the various parameters such as the size, shape, type, nanoparticle load, Re, type of base fluid used, and the tube geometry could affect the HTC of the TiO_2_ nanofluids. One study [19] carried out an experimental analysis of a heated steel surface that was cooled by the laminar nanofluid jets compared to water jets. They studied the effects of three TiO_2_ nanofluid concentrations, i.e., 0.1%, 0.5%, and 1% (w/v), to analyze the jet velocity of the TiO_2_-based nanofluid on the cooling rate. The researchers compared the cooling rate of the Al_2_O_3_, SiO_2,_ and TiO_2_ nanofluids, with the same nanoparticle concentration and velocity. The experimental cooling curves helped to determine the total heat flux using a 1D finite volume process that was based on the transient inverse heat transfer model. The results showed that the heat transfer rate was significantly increased if the researchers used the nanofluid jets compared to the water jets. An increasing TiO_2_ nanoparticle concentration in the nanofluids increased the critical heat flux (CHF). The nanofluid-coated jet-cooled surfaces showed a faster shift from the film boiling to the transition boiling.

### 1.2. Nano Coating Applications

The surface coating helps in improving the heat transfer rate and the performance of several thermal systems. An experimental analysis of the carbon nanotube-coated rectangular brass-extended surfaces was carried out by [20]. They used the finned surface for a free convection cooling of the internal combustion engines and other similar electronic kits. This study also measured the surface temperature and estimated the convective heat transfer rate. Furthermore, the researchers stated that the system performance could be noticeably improved by increasing the heat transfer rate of the extended surfaces. Based on the above-mentioned requirements, the researchers coated the brass surfaces with the carbon nanotubes. Finally, the researchers compared the performances of the rectangular-coated and the non-coated brass fins. The heat transfer rate showed a mean 12% increase for the rectangular, carbon nanocoated brass fins.

Furthermore, Nagarani et al. investigated the increase in the heat transfer rate on elliptical, AISI stainless steel 304 (AISI SS 304), annular fins, which were or were not nanocoated with the multiwalled carbon nanotubes (MWCNTs). They compared various parameters, such as the thermal conductivity, temperature distribution, and convective HTC, while estimating the differing heat inputs. Their results showed a 7% increase in the convective HTC for the coated fin, a 6.2% increase in the fin-shaped tube efficiency, and a 21.8% increase in fin effectiveness [21].

Ray et al. investigated and estimated the nucleate pool boiling heat transfer rate of R134a on a TiO_2_-coated surface. They compared their results with those acquired from a plain copper surface and noted a maximal HTC increase when the surface coat thickness was 200 nm, while a minimal HTC increase was seen when the surface thickness was 100 nm. Coated surfaces showed a better HTC compared to the uncoated surfaces owing to their augmented roughness and an increase in the dynamic nucleation site density [22].

Li et al. studied the effects of nanostructure coating on the thermal performance of thermosyphon boiling in the microchannels. They noted that the nanoparticle coating on heating surfaces could significantly increase the heat transfer rate of the micro thermosyphon. The researchers stated that the CuO nanoparticles showed the highest effect on the heat transfer rate. Meanwhile, the optimal nanoparticle mass concentrations, which showed the highest increase in the heat transfer rate, was seen to be 0.75 wt % for Cu and 1.0 wt % for both Al_2_O_3_ and CuO [23].

In their study, Mitrovic and Hartmann [24] used an electrocoating process for developing a new micro-fin structure that consisted of cylindrical fins. They used the R-141b material and noted that an increase in the heat flux helped in maintaining the wall superheat constant. Furthermore, Melendez and Reyes [25] also noted that when they coated the surfaces with the porous metal layer, it helped to increase the boiling HTC for the binary ethanol-water mixture. Tehver et al. also observed that a boiling heat transfer of the R-113 from the porous metal-coated surface was increased when the surface was coated with a porous, honeycomb oxide layer of high thermal conductivity [26].

Azemati et al. investigated the effect of the nanoparticles on the various heat transfer-related problems and the polymeric coatings. In their study, the researchers added three different nano-zirconium oxide concentrations, i.e., 1%, 3%, and 5%, to a polyurethane resin that was then applied to some metallic surfaces. They also measured the thermography and the emissivity coefficient of all samples along the long wavelengths (IR) to determine the radiation HTCs. They noted that when all three concentrations of zirconium oxide nanoparticles were added to a polyurethane resin, the emissivity coefficient and the absorption of the surface coat significantly increased compared to the control samples without nanoparticles [27].

Nithyanandam and Palanisamy used various surface modification processes for conducting effective heat removal. Wettability was a significant parameter in the multiphase heat transfer. The researchers determined the wettability by measuring the water contact angles on the modified and uncoated surfaces. Here, the researchers abraded copper surfaces using three grades of emery sandpaper, 100, 220, and 600, and nanocoated the surfaces for 15, 30, and 45 mins. They improved the wettability of the nano-coated surfaces and the hydrophilic surfaces [28].

In another study, the researchers studied the pool boiling behavior of their nanoparticle-coated surfaces. They nanocoated the surfaces while conducting the nanofluid pool boiling experiments (Al_2_O_3_–water/ethanol mixtures). These nanocoatings significantly improved the critical heat flux. The ethanol nanofluids also led to a uniform surface nanocoat, which was much better than the water nanofluids-based coating [29].

Furthermore, Phan et al. studied the nano-surface coats for modifying the surface topography slightly in order to alter the static contact angle from 22° to 112°. The researchers noted the formation of bubbles on the surface at a low heat flux in comparison to the wetted standard surfaces. In the case of the weakly wetted surfaces (45° < θ < 90°), the HTC was seen to decrease significantly with a decrease in the contact angle. However, in the case of wetted surfaces (θ ⩽ 45°), an opposite effect was noted, wherein the HTC increased with an increased wettability. The researchers stated that an optimized HTC could be obtained if the contact angles were close to 90° and at low contact angles near 0° [30].

To summarize, many studies reported an increase in HTC values. The results indicated that parameters like size, shape, type, nanoparticle load, and type of base fluid used could affect the HTC value of the TiO_2_ nanoparticles. However, some characteristics of heat transfer nanofluids, such as the jet impingement, spray, high pressure, and high temperature, require further investigation. As stated in the above-mentioned review, there is a lack of information about using TiO_2_ nanosolution coating of aluminum surfaces on heat transfer enhancement. Furthermore, there is a clear gap of information about using TiO_2_ nanosolution coating in the twin impingement jet technique and about the effects between the nanosolution coating and the impingement jet process used. Furthermore, this literature review also highlights the opportunities and the study direction for exploring the heat transfer applications of the TiO_2_ nanocoating techniques. In future, these challenges could be overcome by developing the nanoparticle technology [18].

## 2. Experimental Setup and Procedure

### 2.1. Twin Impingement Jet Setup

**Figure 1:**
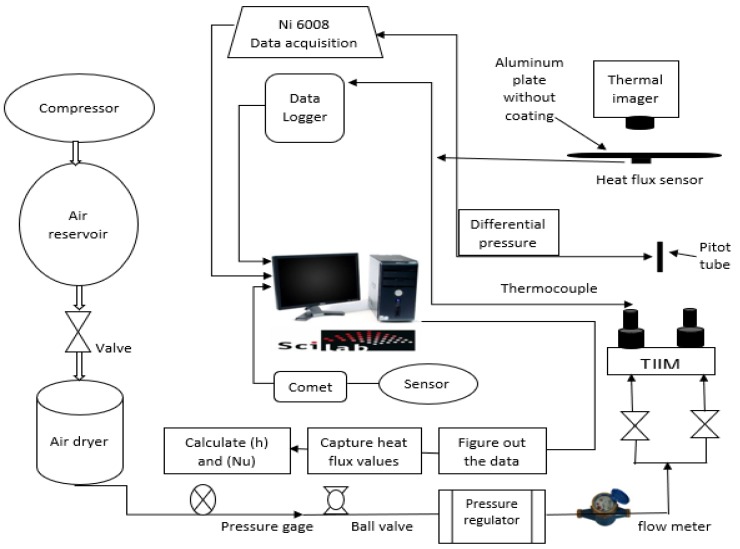
A schematic diagram of the heat transfer test and the thermal imaging setup.

**Figure 2:**
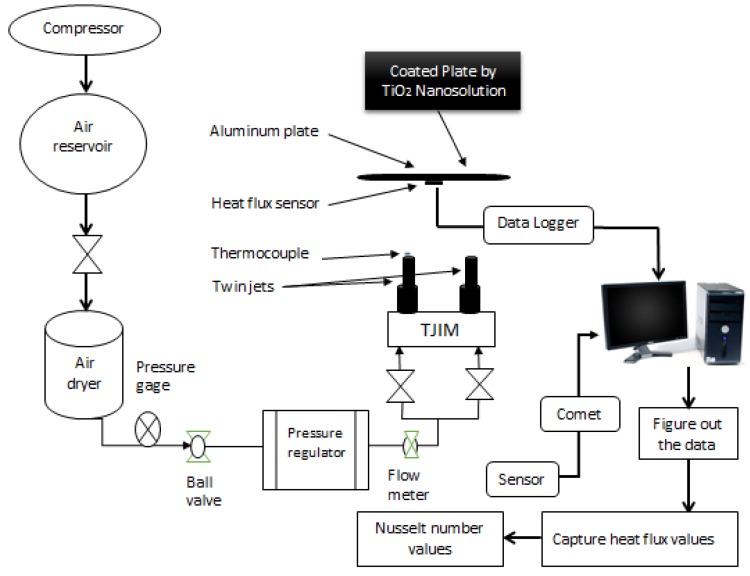
A schematic diagram of the heat transfer test with a TiO_2_-nanocoated surface plate.

Figure 1 and Figure 2 present a schematic representation of the general experimental setup and instrumentation. The experimental procedure was as follows: (1) The researchers set the air flow such that every steady jet could achieve a Re number of 17,000 by estimating the velocity of the twin jet center point at their nozzle exit with the help of a pitot tube; (2) The researchers installed a digital airflow meter anemometer (Dantec Dynamics, Skovlunde, Denmark) in the twin jet impingement mechanism (TJIM) for measuring the velocity and the flow rate of the steady jet flow in the constant temperature mode at 100 °C. The researchers placed a flow meter between the TJIM pipes and the refrigerated air dryer that pass the twin jets. Furthermore, the twin impingement jets were run using the velocity derived from a pitot tube, which was verified using a flow meter. Compressed air (4 psi or 0.275 bar) was supplied from the main compressor and stored in an air reservoir, which was controlled with the help of a ball valve. The researchers used a refrigerated air dryer for eliminating all moisture from this compressed air. We further installed a pressure gauge and a regulator for controlling the air pressure and preventing the fluctuations that could occur due to a cyclic on/off switch of the compressor. We measured the air flow rate with the help of a digital air flow meter (VA 420, CS Instruments, Harrislee, Germany).

**Figure 3:**
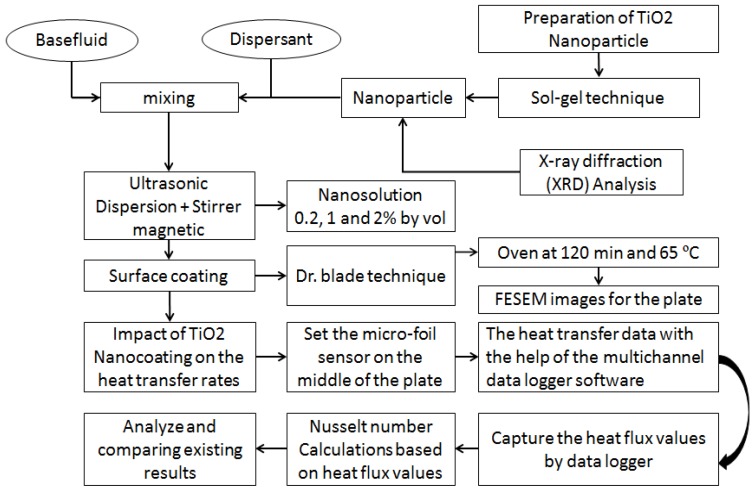
Flow chart of experimental procedure and methodology of twin-impingement jet test setup.

The air could enter the twin jet impingement system through two similar pipelines. A ball valve controlled every pipeline to ensure similar flow characteristics of the twin jets. A square aluminum foil sensor (30 × 30 × 0.4 cm) along with a heat flux-temperature foil sensor were installed on the foil surface using a Kapton tape compound and a high-conductivity heat sink for reducing the effects of the air gap trapped between the aluminum surface and the sensor [2,4]. This foil ensured a flatter impingement surface, which further helped in acquiring a Re number of 17,000 for measuring the heat transfer/unit time (*q*) from a data logger and estimating the convective HTC (*h*) (W/(m²·K)). Forced convection is dominant during the jet impingement heat transfer. The Newton’s law equation can be employed to determine the heat-transfer coefficient (*h*).

(1) $$h=\frac{q}{T_{s}-T_{j}}$$

where *T_s_* represents the surface temperature, *T_j_* signifies the air jet temperature, and (*q*) denotes the amount of heat flux (W/m^2^). The Nu number equation is used to calculate the ratio of convective to conductive heat transfer:

(2) $$\mathrm{Nu}=\frac{hd}{k}$$

where *h* indicates the convective heat-transfer coefficient, *d* represents the pipe diameter, and *k* signifies the fluid’s thermal conductivity. Figure 3 describes the experimental procedure and methodology of the twin-impingement jet test setup.

**Figure 4:**
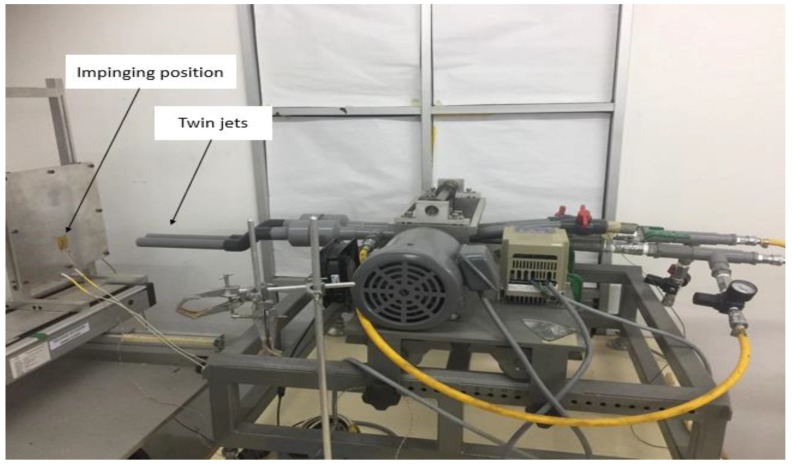
Twin impingement jet mechanism used in the study.

Different models showed a varying nozzle spacing (1, 2, and 3 cm) and a different nozzle-plate distance (1, 6, and 11 cm). This generated nine models, which were investigated further. The researchers used aluminum foil with specific surface and thickness (*L*) dimensions for testing the jet impingement system. The heat flux sensor and thermocouplers were placed in the middle of the aluminum plate surface as shown in Figure 4. The thermal data were collected using a multichannel data logger (GL820, Graphtec Corp., Yokohama, Japan). We also used an infrared thermal imager (Model Ti25, Fluke Corp., Everett, WA, USA) for determining the temperature distribution on the aluminum foil surface. A total of more than 800 samples were recorded by rereading the data that were captured from the data logger to reduce the experimental error in the heat-flux temperature sensor measurements, and the average value was considered; the uncertainty in the measurements of heat transfer was around 2%. Table 1 presents the constant parameters and the values used in this study.

### 2.2. Plate Coating

#### 2.2.1. Preparation of the TiO_2_ Nanoparticals

TiO_2_ nanoparticles were prepared using the sol-gel method described earlier [31]. TiO_2_ (2 g) was dissolved in distilled water (100 mL) by stirring the solution at 60 °C. Then, an aqueous trisodium citrate solution (8% w/v) was added in a dropwise manner at a 0.42 mL/min feed rate to the above reaction mixture for maintaining the titania and trisodium citrate ratio at 1:4. We modified the previous method by increasing the temperature to 60 °C for 4 h to make the solution more stable and homogenous, thereafter, the obtained solution was dried from 80 to 110 °C in a convection oven overnight. This yielded the titania nanoparticles that were used for plate coating, as described below.

#### 2.2.2. Surface Coating

This procedure describes a two-step process for preparing a nanosolution using an ultrasonic bath, magnetic stirrer, and a doctor-blade coating process. The TiO_2_ nanoparticles, prepared above, were dispersed in a deionized water:ethylene glycol (EG) solution (80:20) in three varying concentrations of 0.2, 1, and 2% by volume. This solution was sonicated for 120 min in the ultrasonic bath at a 100 W, the ultrasonic pulse rate was at 40 kHz to ensure a uniform dispersion of the nanoparticles. Thereafter, this solution was poured onto a hot plate using a speed rate at 450 rpm for 15 mins, at room temperature. This sonification process improved the suspension stability since the nanoparticles took a longer time to precipitate (≥120 mins). The TiO_2_ nanoparticles were well dispersed in the water–EG solution before the surface coating process. In addition, the plate was cut into three pieces of 1 × 1 cm^2^, then the aluminum plate surface was cleaned and polished to remove the impurities. Next, the plate was coated using a TiO_2_ nanosolution with three different concentrations via the Dr. Blade method [32]. Finally, the coated plates were placed in the oven at 65 °C for 120 mins.

#### 2.2.3. Analysis of the Nanoparticles and the Coated Surfaces

After the preparation of the nanocoated plates, the researchers identified the phase of the crystalline materials using analytical processes such as X-ray Diffraction (XRD) for determining the unit cell dimensions. This method also enabled them to analyze the composition, structure, and physical material properties. The FESEM analysis used an electron microscope for producing the sample images after scanning the nanocoated surfaces using a focused electron beam. These electrons interacted with the atoms in the specimen and generated a signal, which provided information regarding the sample composition and surface topography.

## 3. Results and Discussion

### 3.1. Structure of the TiO_2_ Nanoparticles

**Figure 5:**
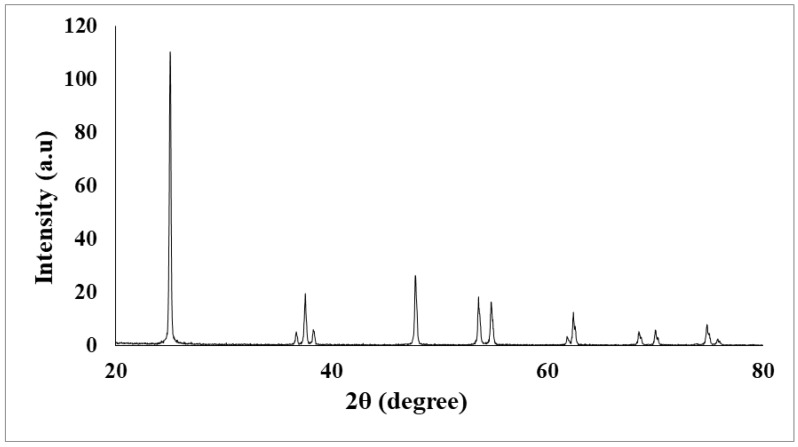
Results of the X-ray diffraction (XRD) analysis.

The researchers used the XRD analysis for determining the XRD patterns of the TiO_2_ nanoparticles that were synthesized using the sol-gel method. The crystallite structure of the TiO_2_ nanocoated plate was calculated using the XRD (Model D8 Advance Bruker AXS X-ray, Karlsruhe, Germany) to identify the crystallite structures of the TiO_2_ nanosolution, the Cu Kα radiation of 1.5406 Å was used in the process. The sample was scanned from 5 to 80° of 2θ at a rate of 0.2°/s. Peaks at the 2θ value were noted at 25°, 37°, 38°, 47°, 54°, 55°, 62°, 69°, and 75° (JCPDS-01-075-2553), which confirmed the formation of the anatase phase. The peaks noted at the different 2θ values showed that these particles were highly pure and crystalline. Moreover, EVA software (version 2, Bruker AXS, Berlin, Germany) was used to evaluate the lattice strain and structure of the sample. The XRD patterns of the samples were analyzed and compared using Joint Committee on Powder Diffraction Standards (JCPDS), the standard from the software library. On the other hand, the use of FESEM (Model: SUPRA 55VP, Carl Zeiss AG, Oberkochen, Germany) was applied to observe the morphology of the TiO_2_ nanocoated plate. Morphological magnification was observed within 100–150 k to obtain clearer images. Figure 5 presents the XRD analysis. 

**Figure 6:**
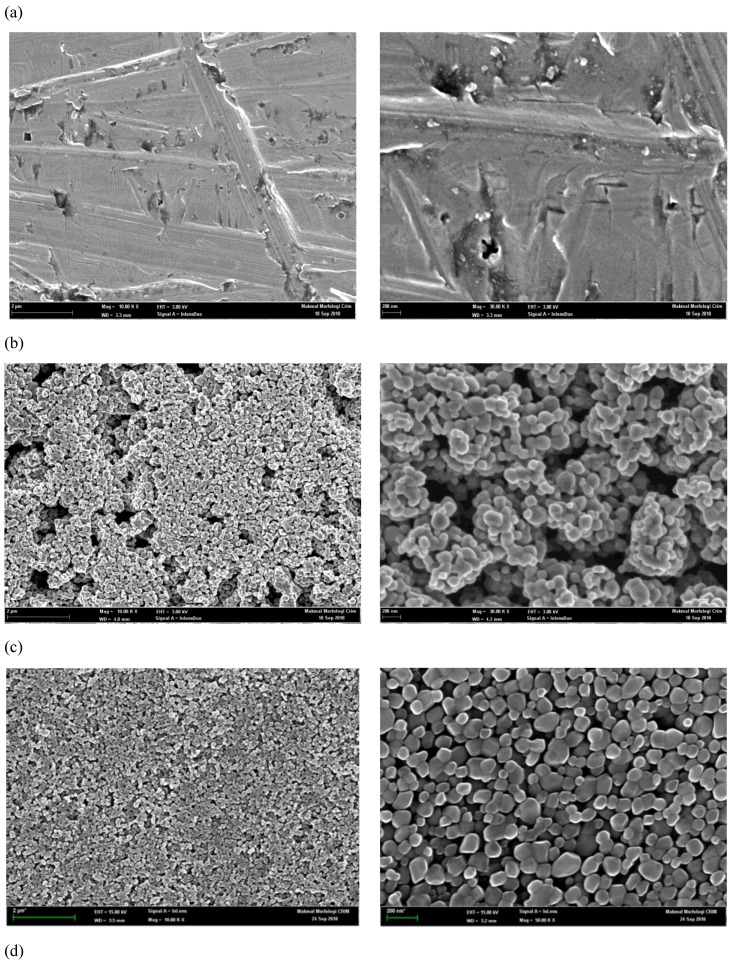

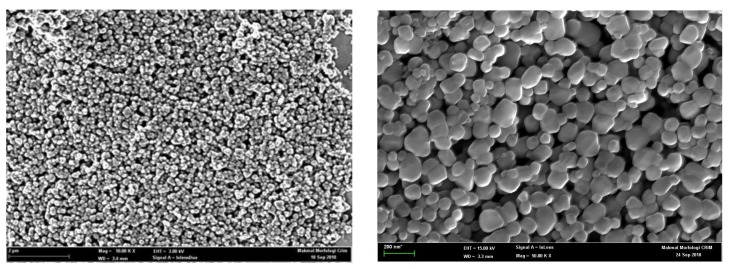
Field Emission Scanning Electron Microscopy (FESEM) micrographs of the surface coated aluminum plates: (**a**) uncoated surface; (**b**) 0.1 M TiO_2_ coat; (**c**) 0.5 M TiO_2_ coat; and (**d**) 1.0 M TiO_2_ coat.

The morphologies of the coated films could significantly affect the electrical and the optical properties of the samples. The researchers also analyzed the surface FESEM micrographs at differing nanosolution concentrations of 0.2, 1.0, and 2.0% by vol as shown in the Figure 6b–d, respectively. The Figure 6a shows the uncoated aluminum plate surface; the FESEM image detected the surface without any change in the surface’s morphology. In addition, Figure 6b–d are micrographs that describe a smooth, densely-packed, homogeneous structure, free of many crystal defects such as cracks, pinholes, and inclusions; the structures are also free of voids. It can be concluded that an increase in the nanosolution concentration vol % affected the grain size and the film thickness. Moreover, an increase in density was observed with an increase in the volume concentration of the solution. It is known that with increasing current density from 0.114 g/cm^2^, 0.117 g/cm^2^, and 0.122 g/cm^2^, the film thickness increases, which results in higher strains in the film [33]. Figure 6 describes the cross-sectional microimages of the various films. The FESEM examinations revealed that the morphology of the sample with a concentration of 0.1 M was in the form of spherical small particles as seen in Figure 6b compared to Figure 6c,d. We believe that the grain growth mechanism was based on the Grain-Rotation-Induced Grain Coalescence (GRIGC) process, described earlier [34]. In their study, the authors of [35] investigated the process, which enabled the grain growth of the nanocrystalline materials and thereafter proposed a novel growth mechanism, i.e., the GRIGC. This model states that the rotation of all grains amongst neighboring grains leads to a coherent interface between the grains (all grains assume a similar crystallographic orientation), which further causes a coalescence of all neighboring grains, by eliminating the common boundaries between the grains, and forms a large single grain. Films formed using a low nanoparticle concentration showed numerous grain clusters of different sizes. However, these grain clusters were converted to single grains at the 1% and 2% vol concentrations. A higher compaction was noted between all grains at 1% compared to 0.2% and 2%. The researchers concluded that the grain clusters had begun their conversion to single grains at 1% (Figure 6c).

### 3.2. Effect of the Various TiO_2_ Nanosolution Concentrations on the Heat Transfer Rate

#### Results of the Interference Zone of Twin Jet Impingement

**Figure 7:**
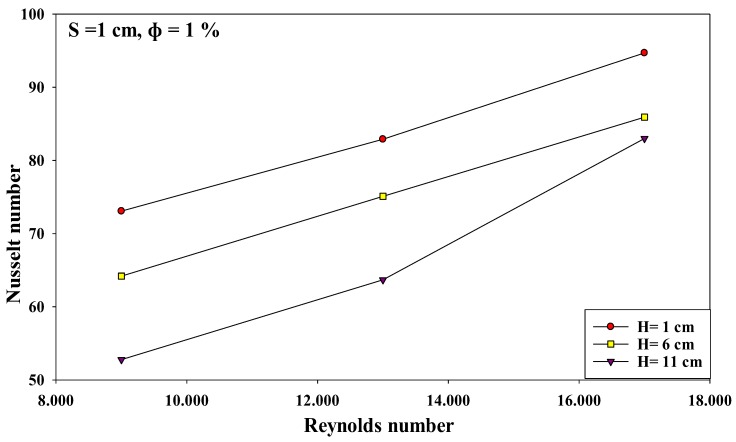
Effect of the Reynolds number and nozzle–plate distance when the nozzle–nozzle distance was 1 cm and other factors were maintained at their optimal levels.

**Figure 8:**
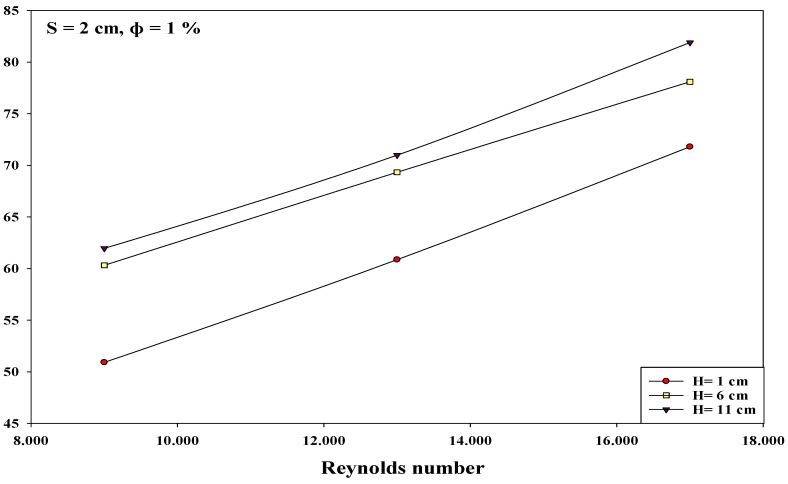
Effect of the Reynolds number and nozzle–plate distance when the nozzle–nozzle distance was 2 cm and other factors were maintained at their optimal levels.

**Figure 9:**
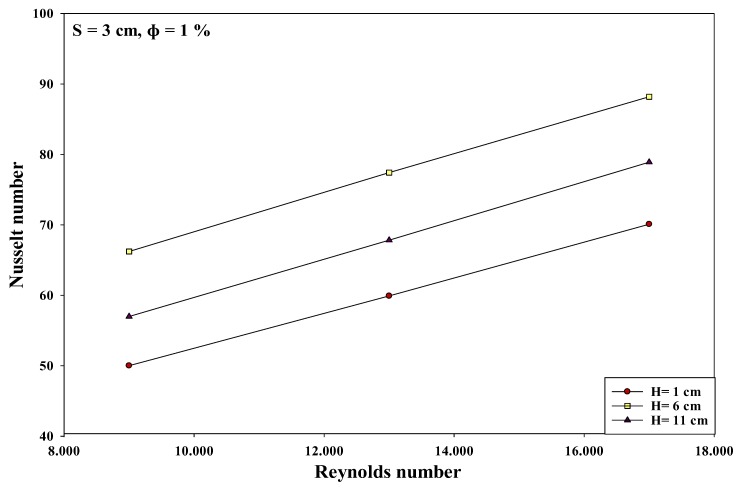
Effect of the Reynolds number and nozzle–plate distance when the nozzle–nozzle distance was 3 cm and other factors were maintained at their optimal levels.

The researchers investigated the interactions between the various input variables for avoiding all conditions that could reduce the heat transfer rates in various applications. All factor interactions were conducted to optimize the nanoparticle concentration at 1%. The researchers studied the two-factor interactions by modifying the values of one of the variables from low to high, and observing its effect on other variables at optimal values. Thus, the effect of one variable depends on the other factor levels if they interact with one another. Figure 7, Figure 8 and Figure 9 describe the interaction between the Reynolds number and the nozzle–plate distance (*H*) at different conditions. Generally, a low nozzle–nozzle distance and low nozzle–plate spacing can improve the heat transfer rate, as described in Figure 7. Furthermore, these distance values also increase with an increasing Reynolds number. The results indicate that the nozzle–plate distance shows a higher effect on the Nusselt number when Re = 9000, compared to when Re = 17,000, specifically when *H* = 6 and *H* = 11 cm. Figure 8 and Figure 9 show the effect of the Reynolds number on the Nusselt number when the distance between the nozzles was 2 and 3 cm, respectively. No effect was noted when the distance was 6 and 11 cm. Thus, the results indicate that the Nusselt number steadily increased with an increasing Reynolds number, for various parameters.

**Figure 10:**
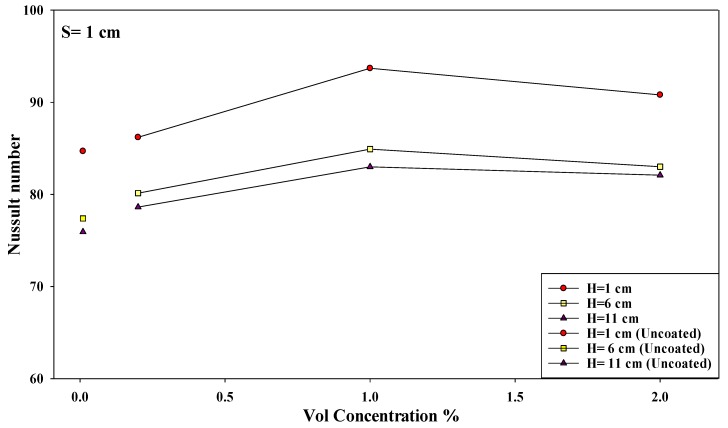
Effect of the nanosolution concentration and nozzle–plate distance when the nozzle–nozzle distance was 1 cm.

**Figure 11:**
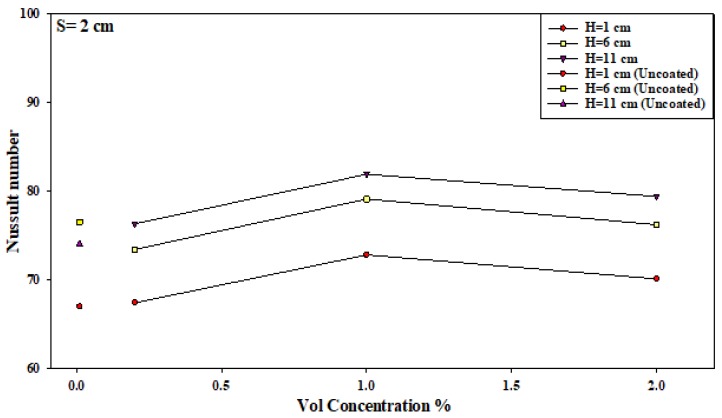
Effect of the nanosolution concentration and nozzle–plate distance when the nozzle–nozzle distance was 2 cm.

**Figure 12:**
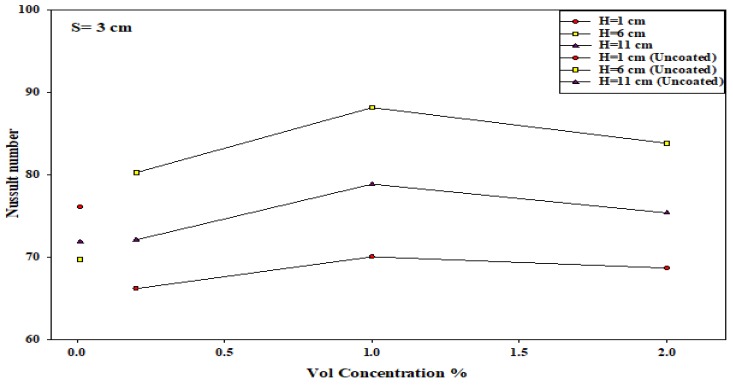
Effect of the nanosolution concentration and nozzle–plate distance when the nozzle–nozzle distance was 3 cm.

Furthermore, the effect of the nozzle–plate distance and the concentration of the nanosolution coating, at other optimal conditions, are described in Figure 10. It can be noted that the Nusselt number increased when the nanosolution concentration increased from 0.2 to 2%; however, it decreased when the concentration increased from 1 to 2%. Furthermore, the Nusselt number also decreased with an increasing nozzle–plate distance. The results showed a sharper decrease in the slope at 0.2% compared to that at 1%, leading to the convergence of the Nusselt number rates at a higher nozzle–plate distance. Thus, an increase in the nanosolution concentration from 0.2 to 1% significantly increased the heat transfer rate at *H* = 1 compared to a higher nozzle–plate distance at the nozzle–nozzle spacing of 2 cm. As shown in Figure 11, the Nusselt number increased with an increasing nozzle–plate distance, wherein the Nusselt number was the highest at 1% concentration, which decreased at 0.2 and 2% concentrations. Figure 12 showed that the Nusselt number was the highest when the nozzle–plate distance was 6 cm. There was an enhancement in the heat transfer rate for the surface coated by the TiO_2_ nanosolution in most cases compared to uncoated surfaces, as shown in the Figures below.

**Figure 13:**
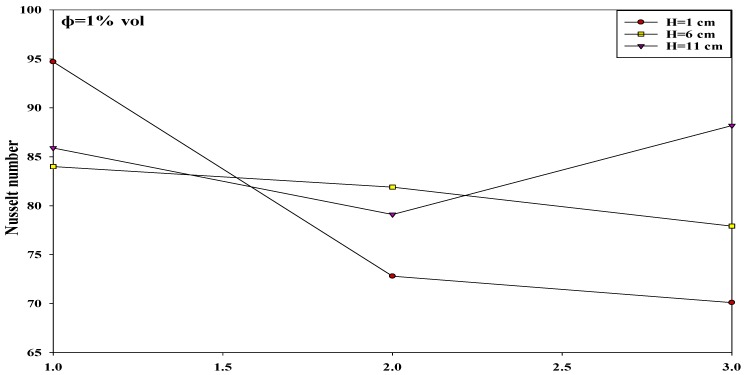
Effect of nozzle–nozzle spacing (*S*) and the nozzle–plate distance (*H*) when other factors were maintained at their optimal levels.

**Figure 14:**
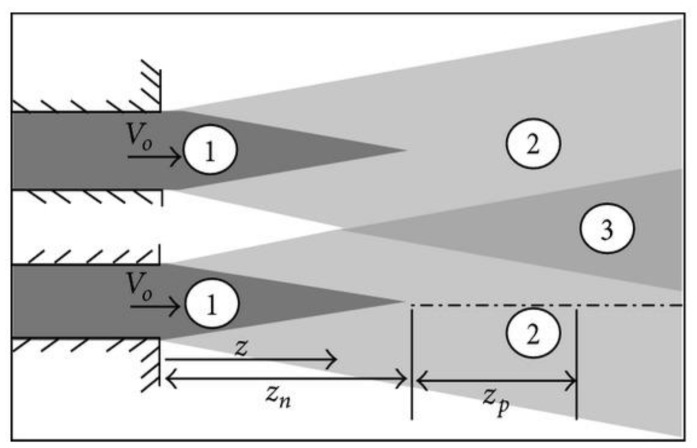
Twin jet configuration.

Figure 13 describes the effect of the nozzle–nozzle distance and the nozzle–plate spacing when other factors were maintained at their optimal levels. In general, an increase in the nozzle–plate distance increased the Nusselt number, until it reached a peak level of *H* = *S* = 1 cm, which decayed with an increasing nozzle–nozzle distance. The twin jets at a distance of 1 cm led to a higher heat transfer rate compared to the values at *H* = 11 cm. On the other hand, at the *H* = 6 cm, the peak point was present at the nozzle–nozzle distance of 3 cm, which decreased at the nozzle–nozzle distances of 1 and 2 cm. Furthermore, at the nozzle–plate distance of 11 cm, a higher Nusselt number was noted at the nozzle–nozzle distance of 1 cm, which decreased with increasing nozzle–nozzle spacing. When the nozzle–nozzle spacing = 1 cm, fluid flow mixing reached a maximum value at the nozzle–plate distance = 6 cm (*Z_P_*) compared with 1 and 11 cm, as shown in the Figure 14. However, convergence of the Nusselt number rates was noted at a lower nozzle–nozzle distance. 

### 3.3. Infrared Thermal Imaging

**Figure 15:**
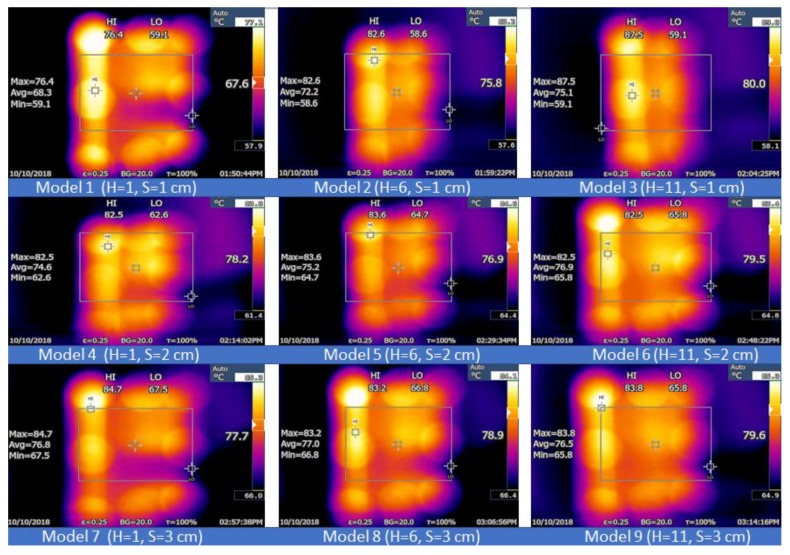
Thermographic distributions of the aluminum flat plate that present Models 1 to 9 at Re = 17,000.

**Figure 16:**
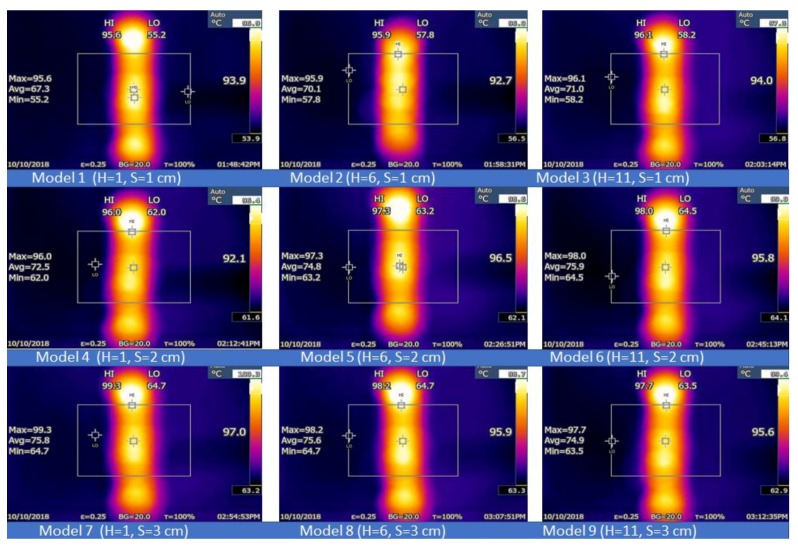
Thermographic distributions of the aluminum flat plate that present Models 1 to 9 at Re = 17,000.

The infrared thermal imaging technique helps in visualizing and enhancing the various physical phenomena in different research fields such as thermal physics, mechanics, optics, electromagnetism, and radiation physics [36]. If the twin jets impinge on the hot aluminum plate, this plate cools and displays specific temperature distribution patterns based on the characteristics and the configuration of the jets. Figure 15 describes the temperature distribution pattern of the impingement plate for various cases at Re = 17,000. All images are labelled with their three different temperature values (max, min, and mean). Nine models for the different steady jet cases are described in the Figure. Model 1 generated a high temperature of 67.4 °C and a low temperature of 59.1 °C when *S* = *H* = 1 cm. Furthermore, nine images were captured at the side plate view to determine the effect of the thermal boundary. Figure 16 presents nine models describing the temperature distribution pattern of the impingement plate at Re = 13,000. The results indicate that the temperature was distributed on the twin impingement jets, which represented the stagnation areas formed when the jet walls clashed together, presenting the impingement effect of the circular, and hot twin jets. All results presented similar thermal and flow characteristics of these twin jets. A higher temperature rate was noted for a steady jet scenario based on the high jet flow rates. Model 1 showed a high temperature of 95.6 °C and a low temperature of 55.2 °C when *S* = *H* = 1 cm. For a convective heat transfer, an increasing thermal boundary decreased the convective HTC rate. Several researchers have aimed in the past to reduce the thermal boundary thickness to improve the convective HTC. Many structures (such as coil inserts, interrupts, surface roughness, etc.) have been developed on the heat transfer surface to improve the HTC, thereby increasing the turbulence and decreasing the thermal boundary thickness, which further increases the HTC value. Analytical results showed a good consistency, indicating the rationality of the model. 

### 3.4. An Improvement in the Heat Transfer Characteristics

In this study, the researchers hypothesized that the heat transfer rate could be significantly improved at an interference zone of the twin impingement jets due to the impingement effect of the jets. This hypothesis was further investigated by conducting several experiments for testing the twin jet heat transfer rate at particular Reynolds number. Based on the results of the flow characteristics, a higher velocity increased the turbulence intensity. Furthermore, based on earlier parametric results, the Reynolds number of 17,000 was seen to be the optimal value that generated the maximal Nusselt number. Hence, the subsequent tests were completed at Re = 17,000 to investigate the increase in the heat transfer rates. Firstly, the researchers studied the effect of the twin impingement jets on the Nusselt number, derived from the above experiments. Furthermore, they also studied the effect of the nanoparticle concentration on the Nusselt number, and also the effects of the jet configurations on the heat flux factor. These tests presented a clear perspective regarding the evaluation of the heat transfer enhancement because of the twin impinging jets. The Nusselt number showed a normalized behavior when the nozzle–plate distance varied from 1 to 11 cm for the twin jets. Figure 7, Figure 8, Figure 9, Figure 10, Figure 11, Figure 12 and Figure 13 show that the Nusselt number varied at the center of the interference zone for the twin jets for the various scenarios. The researchers investigated the effects of the various nanosolution concentrations of 0.2, 1, and 2% at three differing Reynolds numbers (9000, 13,000, and 17,000). It can be seen that the 1% nanosolution concentration showed a higher Nusselt number than the other concentrations. However, at *H* = 1 cm, a better performance was achieved by the steady jet than the others at *S* =1 cm. Hence, two wall jets that were generated by the twin jet impingement on the hot aluminum plate were seen to be the sole source of the cooling air at the secondary stagnation point that was located between these twin jets. It was noted that the heat removal activity from the major stagnation point could be significantly affected after the removal of the heat from the secondary stagnation point. Earlier studies indicated that the steady single jet generated a better heat transfer rate compared to that of the pulsating jet at the stagnation point [37]. A velocity increment was noted at the center line of the interference zone, wherein an increasing velocity led to an enhanced heat transfer and a higher Nusselt number [37,38,39,40,41]. Table 2 illustrates the enhancement ratio of the TiO_2-_nanocoated surface plate in different models.

## 4. Conclusions

In this study, the researchers investigated the convective heat transfer rate of TiO_2_ nanoparticles dissolved in the deionized water (DI)-EG solution. They conducted experiments in a turbulent flow regime. Hence, the researchers investigated the basics of the fluid flow and the heat transfer rate, while highlighting the significance of developing nanotechnology-based research for several heat transfer-related applications. The researchers conclude that the various results derived in this study explain the effects of the various models on the twin impingement jet-based heat-transfer characteristics that enhance the performance of many industrial and engineering applications, and the following conclusions were made:

1. The characteristics of other heat transfer types of nanoparticles such as jet impingement with nanosolution are interesting and worth exploring. 

2. The researchers evaluated the effects of conventional variables such as Re, *H*, and *S* in combination with the novel parameter nanosolution concentration to generate different fluid mixing levels at the stagnation points.

3. Heat transfer characteristics can be enhanced by considering the optimal levels of the influential factors and by selecting a suitable impingement system.

4. The heat transfer rate was directly proportional to the Reynolds number.

5. Nanoparticle structure and nanocoating surfaces were investigated by X-ray diffraction analysis (XRD) and Field Emission Scanning Electron Microscopy technique (FESEM).

6. All the films exhibited a densely packed structure that was smooth, homogeneous, and uniform; the particles were crystallized and had high purity.

7. Nanoparticle concentration increased the heat transfer rate, which restricted the nanosolution concentration.

8. The TiO_2_ nanoparticles dispersed in the water-EG solution noticeably increased the convective heat transfer rate.

9. The highest rate of enhancement in the Nusselt number was around 26%, which was achieved by using TiO_2_ nanocoating.

10. The researchers believe that particle migration was responsible for this increase, which led to a non-uniform distribution of the viscosity and thermal conductivity, which reduced the thermal boundary thickness.

11. Lower nozzle–nozzle and nozzle–plate distances led to a higher heat transfer rate.

## 5. Future Scope

Nanofluid solutions have a large scope in the future of various engineering, industrial, and research-based fields. They can be used in various applications, such as surface coating, engine cooling, nuclear systems, microchips, electronic devices, thermal storage, biomedical devices, the petroleum industry, transportation, defense and space applications, environmental remediation, etc. Surface coating is a vital application that improves the performance and the heat transfer rate of several thermal systems. The nanofluid-based applications of the impingement jet processes are necessary for developing novel devices and require investigation. In the past, researchers have compared the performance of the TiO_2_ nanoparticles with other nanoparticle surface coatings (Al_2_O_3_, Zn, CNT, Cu, etc.) with regards to the flow and heat transfer rate characteristics in various applications. In future, researchers must develop novel nanofluids, such as complex nanofluids with many polymer additives and biological nanofluids, using many computational and experimental processes. A lack of information exists regarding changes in heat transfer rates in the various nanofluids due to jet impingement, spray, higher pressure, and temperature, etc., and this needs further investigation. The above-mentioned challenges have presented some study directions and opportunities for investigation of the application of the heat transfer rate of TiO_2_ nanotechnology.

## Figures and Tables

**Table 1:** Values of the constant parameters.

Constant Parameter	Value
Nozzle-to-plate distance	1 to 11 cm
Nozzle-to-nozzle spacing	1 to 3 cm
Reynolds number	17,000 to 9,000
Ambient temperature	24 °C
Aluminum plate temperature	100 °C
Nanosolution concentration (vol %)	0.2, 1, 2
Background temperature	25 °C
Transmission	100%

**Table 2:** Enhancement ratio of TiO_2_-nanocoated surface plate.

Model	TiO_2_ Nanosolution Concentration by (w/v)	Nu Values (with Coating)	Nu Values (without Coating)	Enhancement Ratio (%)
M1	0.2%	86.2	84.7	10.6
	1%	93.7		
	2%	90.8		
M2	0.2%	80.14	77.4	9.7
	1%	84.92		
	2%	83.01		
M3	0.2%	78.63	75.94	9.2
	1%	83		
	2%	82.1		
M4	0.2%	67.4	66.97	8.7
	1%	72.8		
	2%	70.1		
M5	0.2%	73.4	76.51	3.3
	1%	79.1		
	2%	76.2		
M6	0.2%	76.3	74.03	10.6
	1%	81.9		
	2%	79.4		
M7	0.2%	66.24	76.13	−7.9
	1%	70.1		
	2%	68.73		
M8	0.2%	80.32	69.77	26.6
	1%	88.19		
	2%	83.87		
M9	0.2%	72.16	71.93	8.7
	1%	78.91		
	2%	75.42		
